# Effects of Asymmetric Nuclear Introgression, Introgressive Mitochondrial Sweep, and Purifying Selection on Phylogenetic Reconstruction and Divergence Estimates in the Pacific Clade of *Locustella* Warblers

**DOI:** 10.1371/journal.pone.0122590

**Published:** 2015-04-07

**Authors:** Sergei V. Drovetski, Georgy Semenov, Yaroslav A. Red'kin, Vladimir N. Sotnikov, Igor V. Fadeev, Evgeniy A. Koblik

**Affiliations:** 1 Department of Natural History, Tromsø University Museum, University of Tromsø—The Arctic University of Norway, Tromsø, Norway; 2 Thematic Group on Bird Ecology, Institute of Systematics and Ecology of Animals of Siberian Branch of Russian Academy of Sciences, Novosibirsk, Russia; 3 Department of Ornithology, Zoological Museum of Moscow State University, Moscow, Russia; 4 Kirov City Zoological Museum, Vyatka, Russia; 5 Department of Collections, State Darwin Museum, Moscow, Russia; Instituto de Higiene e Medicina Tropical, PORTUGAL

## Abstract

When isolated but reproductively compatible populations expand geographically and meet, simulations predict asymmetric introgression of neutral loci from a local to invading taxon. Genetic introgression may affect phylogenetic reconstruction by obscuring topology and divergence estimates. We combined phylogenetic analysis of sequences from one mtDNA and 12 nuDNA loci with analysis of gene flow among 5 species of Pacific *Locustella* warblers to test for presence of genetic introgression and its effects on tree topology and divergence estimates. Our data showed that nuDNA introgression was substantial and asymmetrical among all members of superspecies groups whereas mtDNA showed no introgression except a single species pair where the invader's mtDNA was swept by mtDNA of the local species. This introgressive sweep of mtDNA had the opposite direction of the nuDNA introgression and resulted in the paraphyly of the local species' mtDNA haplotypes with respect to those of the invader. Тhe multilocus nuDNA species tree resolved all inter- and intraspecific relationships despite substantial introgression. However, the node ages on the species tree may be underestimated as suggested by the differences in node age estimates based on non-introgressing mtDNA and introgressing nuDNA. In turn, the introgressive sweep and strong purifying selection appear to elongate internal branches in the mtDNA gene tree.

## Introduction

Species' geographic ranges are dynamic and experience changes that can range from steady, slow contractions or expansions, to rapid, dramatic changes caused by crossing biogeographic barriers, geologic events, or human impact on species and their environment. For example, Plio-Pleistocene climatic oscillations and resultant periodic glacial advances had a profound impact on geographic ranges and genetic structure of arctic, boreal, and temperate organisms across the Holarctic. These climatic oscillations reduced ranges of some taxa to small refugial areas or even drove them to extinction. Ranges of other taxa experienced periodic dramatic contractions followed by rapid expansions into newly available habitats [1–4]. Isolation in small refugia and rapid spatial expansion decrease the genetic diversity of populations and facilitate divergence among populations that survived the range contraction part of the cycle in different refugia [5].

Frequently, previously isolated populations come into contact with each other. If these populations are not completely reproductively isolated, these contacts result in the formation of a hybrid zone and genetic introgression. Both simulation and empirical studies have shown that under selective neutrality, the introgression should be asymmetric and in the direction from the earlier arrived population (local taxon) into the more recently arrived one (invader) [5, 6]. In other words, the hybrid zone should be moving in the opposite direction to the apparent gene flow between populations. There are three reasons for this counterintuitive effect of introgression identified by Excoffier *et*. *al* [5]. First, there is a progressive dilution of the invader's genome by the local population's genome or the demic diffusion [7]. The front of the invader's wave is composed of individuals with admixed genomes. Second, the demography of the invader and local taxon are different: the invader's population grows where as the local population is stable or, more likely, declines in the recently invaded areas. Therefore, the loss of local alleles due to genetic drift is less likely from the invader than the loss of the invader's alleles from the local taxon. Finally, local alleles introgressed into the invader can "ride the wave" and increase in frequency due to genetic drift in a growing population and continuous supply of new copies through hybridization.

A static hybrid zone should indicate selection against hybrids [8] and movement of some loci in the opposite direction from the rest of the genome (from an invader to a local population) should be indicative of the selective advantage of invader's alleles [5, 6]. These studies also suggest that under selective neutrality, parts of the genome associated with the least dispersing sex should experience greater introgression than parts associated with the most dispersing sex. This is because the greater supply of genes associated with the most dispersing sex dilutes or even swaps the alleles introgressing from a local taxon into the invader, whereas genes associated with the least dispersing sex are supplied at lower rates that allows the introgressing local alleles to reach a higher frequency in the invader [5]. In the case of birds that, with a few exceptions, have a female-biased dispersal [9], mtDNA and W chromosome should have lower introgression than Z chromosome or autosomes. That was true in all 16 case studies of birds and insects with female-biased dispersal examined by Excofier *et al*. [5].

Genetic introgression can affect reconstructions of phylogenetic relationships among taxa in a number of important ways. An introgressive sweep (or a combination of extensive introgression and limited sampling of individuals) can alter tree topology if the introgression occurs between non-sister taxa. Heterogeneous introgression across loci can produce conflict in phylogenetic signal of different parts of the genome. Introgression can also shorten external branches on a tree but lengthen internal ones and therefore impact divergence time estimates accordingly [10].

Although long underappreciated, genetic introgression is common in birds [10]. Interestingly, there are many avian examples of mtDNA introgression in birds despite that maternally inherited mtDNA should be less likely to introgress in birds than nuDNA in the absence of selection [5, 10] due to female-biased dispersal [11] and possible additional effects of Haldane's rule [12].

To elucidate effects of introgression on phylogenetic reconstruction and gene flow estimates we use nucleotide sequences of the mtDNA ND2 gene and 12 nuclear introns to reconstruct phylogenetic relationships within the Pacific clade of *Locustella* warblers and to determine the intensity and directionality of the gene flow among species within these groups.

The Pacific clade of grasshopper warblers (Aves: *Locustella*) consists of three groups of closely related taxa (superspecies) with uncertain taxonomic status: *fasciolata* group, *certhiola* group, and *pryeri* group [13, 14]. The *certhiola* group consists of three currently recognized species: *certhiola*, *ochotensis*, *and pleskei* [15]. *Certhiola* breeds on the Asian mainland from Western Siberia to the west coast of Sea of Okhotsk and Sea of Japan [16]. *Ochotensis* breeds on Hokkaido, Sakhalin, and Kuril Islands, Kamchatka peninsula, and west coast of Sea of Okhotsk from 49°N to 60°N [16]. Along the Sea of Okhotsk coast, ranges of *certhiola* and *ockhotensis* overlap. A high proportion of phenotypically intermediate individuals has been reported from this area of overlap and neighboring Sakhalin Island raising the possibility of extensive hybridization between these species [17]. *Pleskei* breeds on small islands and islets off Honshu and Kyushu Islands, Korean Peninsula, and in the Peter the Great Bay south of Vladivostok, Russia [16]. The over-water distance separating some islets inhabited by *pleskei* in the Peter the Great Bay from the coast inhabited by *certhiola* is only 2 km.

The *certhiola* group is sister to the single currently recognized species *pryeri*, which breeds on Honshu Island and in easternmost China. The *fasciolata* group consists of two currently recognized species: *fasciolata*—the Asian mainland species breeding from southwestern Siberia to the southern shore of the Sea of Okhotsk and Sea of Japan coast and *amnicola*—breeding on Sakhalin, Hokkaido, and Kuril Islands. Although these two species' ranges do not overlap, Nevelskoy Strait, separating Sakhalin Island from the Asian mainland and the ranges of these species, is only 7 km wide.

There appears to be a conflict between phylogenetic signals of mtDNA and nuDNA in the *certhiola* group. Phylogenetic analyses that used only mtDNA—ND2 [13] and cytochrome-*b* (cyt-*b*) genes [14] to reconstruct relationships among *Locustella* warblers yielded the same tree topology that suggested paraphyly of *pleskei* with respect to *ochotensis* with *certhiola* being more distantly related to them. The analysis of unphased, concatenated sequences of four nuclear introns obtained from a few individuals sampled primarily on wintering grounds or along migratory routes suggested that *ochotensis* and *certhiola* were sisters and *pleskei* was more distantly related to them [14].

It took 40 years from the description of *amnicola* as a distinct species [18] to its recognition by the international community [19]. Prior to 2012, *amnicola* was considered a subspecies of *fasciolata*. Neither phylogenetic study of *Locustella* [13, 14] included multiple individuals of both species from the *fasciolata* group and, thus, did not sufficiently test the monophyly of *fasciolata* and *amnicola*, although in both studies, mtDNA genetic distances between conspecific individuals appear to be much shorter than distances between individuals that belong to their sister species.

Subtle phenotypic differences, parapatric breeding ranges, suspected extensive hybridization at least in one species pair and apparent mito-nuclear conflict in phylogenetic signal in the other make Pacific *Locustella* warblers an excellent group to study genetic introgression between closely related taxa. Therefore, the goal of our study is to test the hypothesis predicting that 1) introgression is common among closely related taxa, 2) nuclear loci introgress more than mtDNA, 3) introgression is predominantly unidirectional, 4) mito-nuclear topological discordance results from an introgressive sweep of mtDNA.

## Material and Methods

### Ethics Statement

This study did not require ethical approval in our institutions because we used samples loaned to us by public museums (State Darwin Museum: IVF0945, IVF0954, SVD4456, EVN0947, EVN0952; University of Washington Burke Museum: UWBM 43834, UWBM 43835, UWBM 43855, UWBM 44413, UWBM 44414, UWBM 44427, UWBM 44428, UWBM 47071, UWBM 47174, UWBM 47180, UWBM 47181, UWBM 58398, UWBM 58399; Yale Peabody Museum: YPM139893, YPM139895, YPM139901, YPM139954, YPM140020, YPM140105, YPM140212; Zoological Museum of Moscow State University: RYA2873, RYA3032, RYA3070, RYA1015, CBH2807, RYA1836, RYA1837, RYA1535, CBH2983, CBH4178, CBH4199, CBH4219, CBH1437, CBH1455, SGA1417, SGA1418, P1121999, P1311999, ZMMU1161999, ZMMU3432000, CBH0622, RYA0664, CBH0654, RYA0830, CBH0509, SGA0899, SGA0943, RYA3074, RYA3068, CBH2305, CBH2363, RYA1539, RYA1547, RYA1611, RYA1660, RYA1661, CBH0743, CBH0744, CBH0745, CBH0746, CBH3537, CBH3538; S1 Table) who comply with relevant regulations for acquisition and curation of their collections.

**Fig 1:**
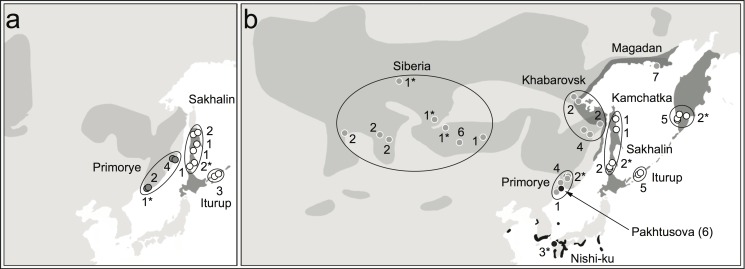
Species ranges, sampling regions, localities and sample sizes for fasciolata species group (a: dark range white circles = *amnicola*, light range dark gray circles = *fasciolata*) and certhiola species group (b: light gray range with dark gray circles = *certhiola*, dark gray range with white circles = *ochotensis*, black range with black circles = *pleskei*). * indicates samples for which only mtDNA ND2 gene sequence data were available.

We obtained a total of 67 tissue samples of *Locustella* warblers from museum collections (Fig. 1; S1 Table). These samples are primarily breeding adults (n = 60). Five local juvenile *fasciolata* and two *certhiola* were used due to lack of adult samples from respective localities. To avoid misrepresenting allele frequencies in our data, we reduced the possibility of sampling juveniles with their siblings and parents by using juveniles collected at different localities or on different dates (at least 5 days apart) than any other bird of the same species. We sampled two species groups (*fasciolata* and *certhiola*) within the Pacific clade of *Locustella* warblers [13, 14]. The *fasciolata* group—was represented by *fasciolata* samples from Primorye (n = 6) and by *amnicola* samples from Sahalin Is. (n = 5) and Iturup Is. (n = 3). The *certhiola* group was represented by *certhiola* samples from Siberia (n = 13), Primorye (n = 5), Khabarovsk (n = 8), and Magadan (n = 7), by *ochotensis* samples from Sakhalin Is. (n = 4), Iturup Is. (n = 5), and Kamchatka (n = 5), and by *pleskei* samples from Pakhtusova Is. off the coast of Primorye (n = 6; Fig. 1).

Total genomic DNA was extracted from tissue samples using the JETQUICK Tissue DNA Spin Kit (Genomed, Loöhne, Germany) according to the manufacturer’s instructions. For all samples, we attempted to sequence the complete mtDNA ND2 gene (1041 bp) and 12 nuclear introns located on different chromosomes (S2 Table). With a few exceptions, we obtained sequences of 11 introns for most individuals. For 1 of the 12 introns (NAT15), PCRs failed for most of the members of the *certhiola* group but worked well for the members of the *fasciolata* group (S1 Table). We also used 15 mtDNA ND2 gene sequences of *Locustella* warblers from the two species groups available in GenBank (accession numbers: AY382357—AY382359, AY382363—AY382367, AY382388—AY382394; S1 Table).

PCR fragments were sequenced in both directions on an ABI 3730 Genetic Analyzer (Applied Biosystems Inc., Foster City, CA) at the Macrogen Europe facility. The sequences were aligned automatically in Sequencher 5.0.1 (Gene Codes Corporation, Ann Arbor, MI) and verified manually to ensure consistent alignment of indels.

In heterogametic individuals whose alleles differed in length, the alleles were identified by subtracting the complimentary sequence of the allele without the indel from the double peaks in their chromatogram [20]. Alleles of heterogametic individuals that had the same length but contained multiple nucleotide differences we resolved using PHASE 2.1.1 [21]. We conducted two independent PHASE runs. The first 500 interactions were discarded as burn-in. The following 5000 iterations used a thinning interval of 10.

We used *BEAST 2.0.2 [22] to reconstruct locus-specific (ND2) and multi-locus species tree (nuDNA; site models, clock models and tree models unlinked) and to estimate divergence times among taxa. We used the mean rate of sequence evolution and associated 95% confidence interval (CI) reported for ND2 gene (2.9 x 10^–2^ substitutions/site/Ma; 95% CI 2.4–3.3 x 10^–2^) of Hawaiian honeycreepers (Aves: Drepanidinae) [23]. For nuclear loci, we allowed these rates to be estimated relative to the evolutionary rate of ACO1I9 (3.2 x 10^–3^ substitutions/site/Ma; 95% CI: 2.4–4.0 x 10^–3^; S2 Table) reported for accentors (Aves: Prunellidae) [20] that was originally estimated relative to the ND2 rate of [23].

We used the Bayesian information criterion (BIC) implemented in jModelTest 2.1.2 (Posada 2008) to select substitution models (S2 Table) for the *BEAST analyses. To select the appropriate molecular clock prior, we conducted two independent runs for each locus. In one run, we used a strict clock prior and in the other, a relaxed lognormal clock prior. We then conducted a maximum likelihood ratio test [24] to determine whether the strict clock tree likelihood was significantly worse than the relaxed clock tree likelihood. Because MLRT was not significant (all P values ≥ 0.80) for any of our loci, we report the results of our *BEAST analyses with the strict molecular clock prior.

Three separate MCMC analyses were run for 10^9^ generations with a 5000-generation burn-in and parameters sampled every 10^4^ steps. Independent runs were combined using LogCombiner 2.0.2 [22]. Tracer 1.5 (http://beast.bio.ed.ac.uk/Tracer) was used to determine the effective sample size of each parameter and calculate its mean and 95% highest posterior density (95% HPD) interval. Tree topologies were assessed using TreeAnnotator 2.0.2 [22] and visualized in FigTree 1.3.1 (http://tree.bio.ed.ac.uk/software/figtree/).

We used Migrate 3.5.1 [25] to estimate gene flow among species in each species group. We ran separate analyses of the mtDNA and of all nuclear loci sequences. Each analysis consisted of three independent runs. First runs employed default settings for the population (*θ*) and migration (*M*) parameters. In the last two runs we used estimates of *θ* and M from the first run as starting values. All runs employed Bayesian inference, a different random seed, and varying mutation rates estimated from data. A single long Markov chain consisted of 2.5 x 10^7^ steps with sampling increment of 10^2^ steps and was replicated twice. The number of sampled parameter values was 5 x 10^9^. The number of trees discarded per chain (burn-in) was 2.5 x 10^5^. We used the static heating scheme of 4 chains with temperatures 1000000.00 3.00 1.50 1.00 and swapping interval of 2.

To test whether mtDNA ND2 gene was neutral, we conducted the McDonald–Kreitman test [26] implemented on the following website: http://mkt.uab.es [27]. The HKA test [28] implemented in HKA software (https://bio.cst.temple.edu/~hey/software/software.htm#HKA) was used to test neutrality of nuclear introns. We used two sister species pairs for HKA test: fasciolata/amnicola and certhiola/ochotensis.

## Results

### MtDNA Gene Tree Versus Nuclear Multilocus Species Tree

**Fig 2:**
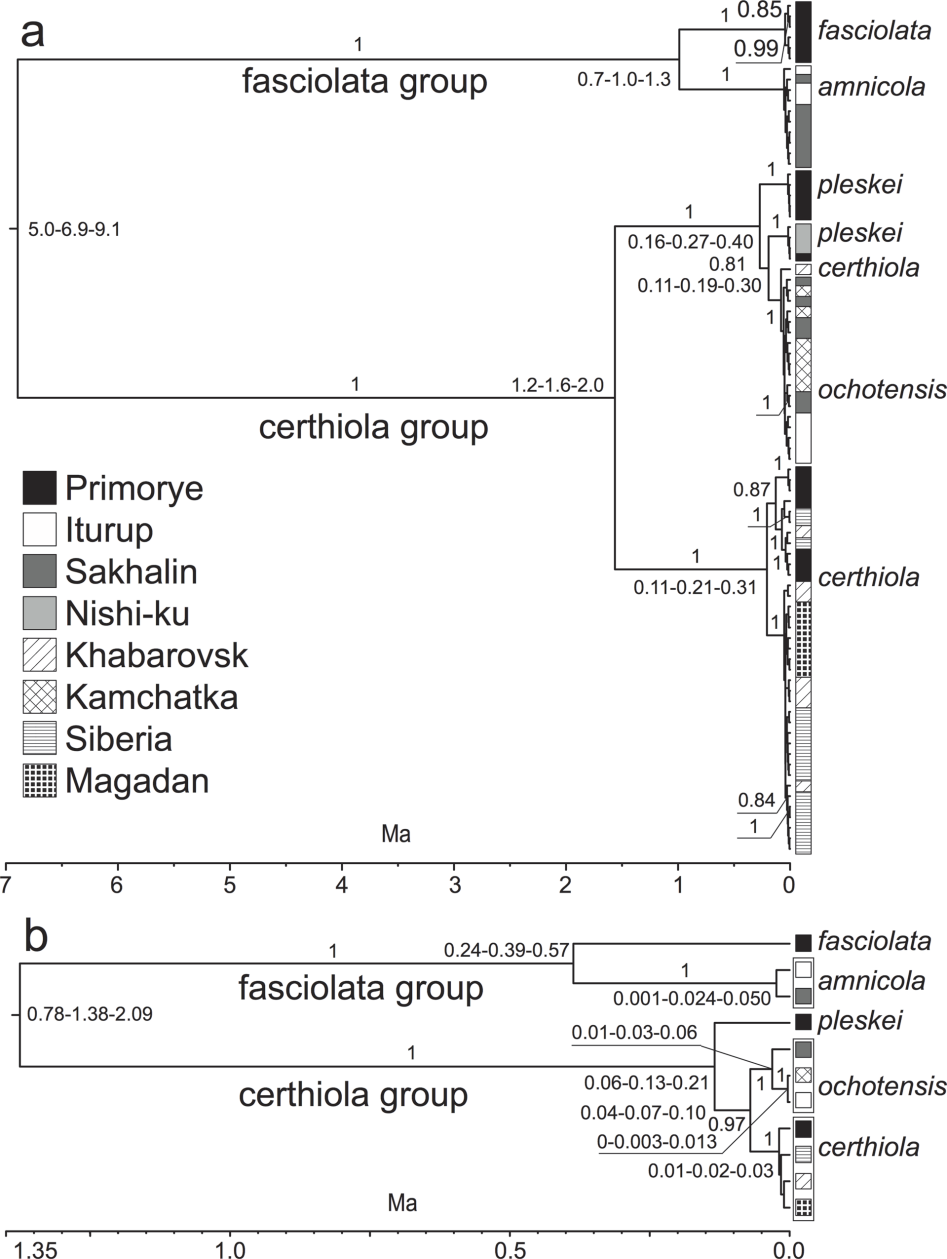
Phylogenetic tree based on MtDNA ND2 haplotypes (a) and multilocus species tree based on nuclear introns (b). Numbers next to branches indicate their posterior probability. The hyphenated numbers next to nodes identify the mode of their ages (middle number) and 95% HPD intervals (lower in front and higher after the mode). The time scale below each tree and node ages are in million years (Ma) before present.

The monophyly of each species group, *fasciolata* and *certhiola*, were strongly supported in the mtDNA ND2 gene tree (both PP = 1; Fig. 2A). The mtDNA divergence between *fasciolata* and *certhiola* groups was estimated at 6.9 Ma (95% HPD interval 5.0–9.1 Ma).

The two species of the *fasciolata* group, *amnicola* and *fasciolata* were reciprocally monophyletic in the mtDNA ND2 gene tree with each species supported by PP of 1 (Fig. 2A). The divergence between these two species was estimated at 1.0 Ma (95% HPD interval 0.7–1.3 Ma). Haplotypes from both *amnicola* localities, Iturup and Sakhalin Islands, were intermixed in the *amnicola* clade.

The McDonald–Kreitman test for *amnicola* and *fasciolata* clades indicated that the ratio of the synonymous to non-synonymous substitutions among polymorphisms within clades (6:2) did not differ significantly from the same ratio among the differences between the clades (38:7; the neutrality index NI = 1.809, P = 0.512). The NI value indicates the presence of mild, not significant, purifying selection—selection against deleterious non-synonymous substitutions.

In the *certhiola* group, none of the currently recognized species was monophyletic in the mtDNA ND2 gene tree (Fig. 2A). This group was divided into two deeply divergent (1.6 Ma, 95% HPD interval 1.2–2.0 Ma) and strongly supported clades (both PP = 1). One clade consisted exclusively of *certhiola* individuals (all but one), whereas the other included all three species of the *certhiola* group. The second multispecies clade was divided into three strongly supported (all three PP = 1) subclades. One subclade included 5 of 6 *pleskei* individuals sampled on the Pakhtusova Is. in Primorye. The second subclade included all 3 *pleskei* sampled on Nishi-ku off the coast of Honshu Is. in Japan and a single *pleskei* individual sampled on the Pakhtusova Is. in Primorye. The third subclade included all *ochotensis* samples and a single *certhiola* collected on the shore of Lake Udyl, 100 km inland from the Pacific coast (Khabarovsk). The latter specimen (VM510, UWBM 58398) is a phenotypic *certhiola* and was collected together with another phenotypic *certhiola* specimen (VM511, UWBM 58399), which had a mtDNA ND2 sequence that fell within the exclusively *certhiola* clade.

The McDonald–Kreitman test for *certhiola* and *pleskei/ochotensis/certiola* clades indicated that the ratio of the synonymous to non-synonymous substitutions among polymorphisms within clades (52:14) was significantly different from this ratio among the differences between the clades (41:3; the neutrality index NI = 3.679, P = 0.040). This indicates the presence of significant purifying selection.

HKA test results found no significant deviations of intraspecific polymorphism and the mean pairwise divergence in both sister pairs from those expected under neutrality (P-values for *fasciolata/amnicola*, 12 loci was 0.082 and for *certhiola/ochotensis*, 11 loci = 0.955). Therefore, neutral evolution of the introns used in our study could not be rejected.

The nuclear multilocus species tree based on complete sequences of 12 introns sampled from different chromosomes provided much better resolution than the mtDNA ND2 gene tree (Fig. 2B) despite widespread lack of lineage sorting across loci (S1 Fig). Similar to the mtDNA tree, the monophyly of each species group, *fasciolata* and *certhiola*, was strongly supported (both PP = 1). However, the divergence between these groups was estimated to be five times more recent (1.4 Ma; 95% HDP interval 0.8–2.1 Ma), than the age estimate based on the mtDNA tree.

The divergence between *fasciolata* and *amnicola* in the multilocus nuclear tree was estimated at 0.4 Ma (95% HPD interval 0.2–0.6 Ma), which is more than twice as recent as the divergence estimated in the mtDNA gene tree. Both *amnicola* populations, Sakhalin and Iturup Islands, were monophyletic (PP = 1) and very closely related—their divergence was estimated at 0.024 Ma (95% HPD interval 0.001–0.050 Ma).

In contrast to the mtDNA gene tree, all three species in the *certhiola* group and their relationships appeared to be well resolved (Fig. 2B). The monophyly of *certhiola* (PP = 1) and *ochotensis* (PP = 1) and their sister relationship (PP = 0.97) were strongly supported and *pleskei* was more distantly related to them. The divergence of *pleskei* from the sister pair of *certhiola* and *ochotensis* was estimated at 0.134 Ma (95% HPD interval 0.069–0.207 Ma), whereas *certhiola* and *ochotensis* diverged half as recently—0.070 Ma (95% HPD interval 0.043–0.103 Ma). Furthermore, the nuclear multilocus tree detected divergence of the Sakhalin population of *ochotensis* from Kamchatka and Iturup birds (PP = 1). This intraspecific divergence was estimated at 0.031 Ma (95% HPD interval 0.013–0.055 Ma).

### Gene Flow Among Species

**Fig 3:**
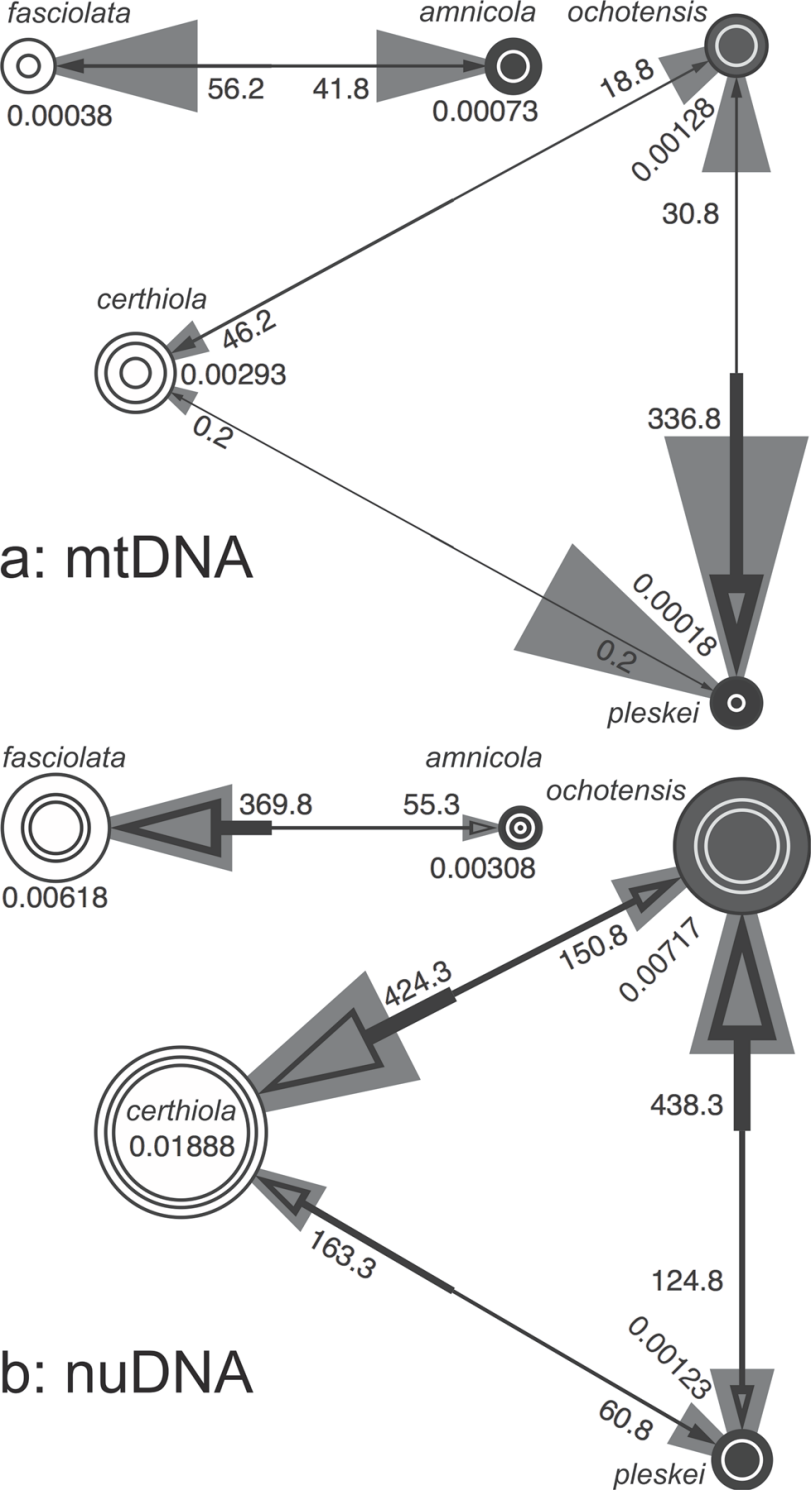
Population (*θ*) and gene flow (*M*) parameter values estimated with mtDNA ND2 haplotypes (a) and multilocus nuclear intron sequences (b). The diameter of the innermost and outermost circles and the thickness of gray arrows represent lower and higher 95% HPD intervals for the parameter values (*θ* and *M*, respectively). The size of the middle circles and thickness of black arrows represent the modes of the parameter estimates. When only two circles are shown or the smaller gray arrow is missing, the lower 95% HPD interval is 0. The numbers next to the circles show mode estimates for the population parameter (*θ*), and the numbers next to the arrows show mode estimates for the gene flow parameter (*M*).

MtDNA ND2 gene sequences provided little information on population sizes or gene flow among taxa. All population size parameter (*θ*) values and all but a single migration parameter (*M*) value had large 95% HPD intervals that included 0 (Fig. 3A). This was true for all *θ* and *M* parameters in the *fasciolata* group. In the *certhiola* group, only the *M* value from *ochotensis* to *pleskei* did not include 0 in its lower 95% HPD interval (*M* = 336.8; 95% HPD interval 67.5–967.5). These results cannot reject the lack of gene flow among all taxa within both species groups except from *ochotensis* to *pleskei*.

Similar to the phylogenetic reconstruction, our nuclear multilocus dataset provided much better resolution in the analysis of gene flow among species. In the *fasciolata* group, insular *amnicola* had half the population size parameter *θ* value (*θ* = 0.00308, 95% HPD interval 0.00110–0.0500) of that for continental *fasciolata* (*θ* = 0.00618, 95% HPD interval 0.00330–0.00955; Fig. 3B). The gene flow parameter *M* from *fasciolata* to *amnicola* was almost 7 times lower (*M* = 55.3; 95% HPD interval 7.0–141.5) than the *M* value from *amnicola* to *fasciolata* (*M* = 369.8, 95% HPD interval 221.0–568.0).

In the *certhiola* group, the population size parameter *θ* value was the lowest for *pleskei* (*θ* = 0.00123, 95% HPD interval 0–0.00285) breeding on small islets, insular/coastal *ochotensis* had an intermediate *θ* value (*θ* = 0.00717, 95% HPD interval 0.00435–0.01025), and continental *certhiola* had the largest *θ* value (*θ* = 0.01800, 95% HPD interval 0.01515–0.02420; Fig. 3B). The gene flow parameter *M* value from *pleskei* to *ochotensis* (*M* = 438.3, 95% HPD interval 227.0–694.0) was 3.5 times greater than the *M* value in the opposite direction (*M* = 124.8, 95% HPD interval 5.5–406.0). The *M* value from *pleskei* to *certhiola* (*M* = 163.3, 95% HPD interval 65.5–318.0) was almost 3 times greater than *M* for the opposite direction (*M* = 60.8, 95% HPD interval 0–289.5). Finally, the *M* value from *ochotensis* to *certhiola* (*M* = 424.3, 95% HPD interval 314.5–813.0) was almost 3 times greater than *M* in the opposite direction (*M* = 150.8, 95% HPD interval 26.0–357.5).

## Discussion

### MtDNA Gene Tree Versus Nuclear Multilocus Species Tree

Although the mtDNA ND2 gene tree strongly supported the monophyly of the two species groups and provided much stronger support for two species within the *fasciolata* group than in previous studies [13, 14], it failed to resolve the relationships among species of the *certhiola* group (Fig. 2A). The use of additional samples of all three species in that group, *pleskei*, *ochotensis*, and *certhiola*, compared to those used in earlier studies [13, 14] did not improve the mtDNA tree resolution but instead further obscured it. In particular, the new mtDNA ND2 gene tree strengthened the support for the paraphyly of *pleskei* in respect to *ochotensis* and indicated that one *certhiola* (VM510, UWBM 58398) carried *ochotensis* mtDNA (Fig. 2A). The latter specimen was collected in the zone of range overlap of the two species where hybridization between them has been suspected on the basis of intermediate phenotypic characters observed in many specimens [17].

In contrast to the mtDNA ND2 gene tree, a species tree based on nucleotide sequences of 12 nuclear introns completely resolved the phylogenetic relationships between and within *fasciolata* and *certhiola* species groups (Fig. 3B). For 3 of 5 species, *amnicola*, *certhiola*, and *ochotensis*, we used multiple regional samples as taxa in the multilocus species tree reconstruction. In all 3 species, the monophyly of conspecific samples from different geographic regions had PP = 1, strongly supporting current species-level taxonomy in both species groups despite the apparent hybridization between *ochotensis* and *certhiola* [17] and nuDNA introgression revealed by our gene flow analyses (Fig. 3B).

Our mtDNA phylogeny supported the presence of mtDNA introgression from *ochotensis* to *certhiola*. One of 38 phenotypic *certhiola* specimens carried *ochotensis* mtDNA (Fig. 2A). This is unlikely to be due to incomplete lineage sorting of mtDNA haplotypes between *ochotensis* and *certhiola*. MtDNA clades are well differentiated (46 fixed substitutions) which is consistent with introgression but inconsistent with incomplete lineage sorting [10, 29–31] and will require a large number of homoplasious mutations.

Similar to the mtDNA ND2 gene tree, our multilocus species tree provided strong evidence for distinct status of *amnicola*. None of the mtDNA ND2 haplotypes (Fig. 2A) was shared by *amnicola* and *fasciolata* and only 3 nuclear alleles across the 12 sampled loci were shared (S1 Fig). In contrast to the mtDNA ND2 gene tree, however, our multilocus species tree suggested that *certhiola* is the sister to *ochotensis* and that *pleskei* is their more distant relative. This clearly indicates the presence of a strong mito-nuclear conflict in the phylogenetic signal. The fact that *ochotensis* mtDNA haplotypes form a low diversity clade (PP = 1) imbedded within *pleskei* suggests an introgressive sweep of *pleskei* mtDNA in *ochotensis*. MtDNA sweep should be suspected in the case of a strong mito-nuclear conflict in phylogenetic signal between mtDNA and nuDNA [10, 31]. In this case an introgressive sweep of *pleskei* mtDNA in *ochotensis* is further supported by the opposite direction of mtDNA introgression to that of nuDNA between these species (Fig. 3) while the expectation under neutrality is either the lack of mtDNA introgression or a its lower level introgression in the same direction as nuDNA introgression [5, 6].

Our multilocus species tree also indicated the presence of intraspecific structure in *ochotensis* where division between Sakhalin and Kamchatka + Iturup samples suggests evolutionary independence of the Sakhalin population from more eastern populations (Kamchatka and Kuril Islands).

The mtDNA gene tree and the multilocus species tree differed significantly in their divergence time estimates. The depth of the mtDNA ND2 tree was 5 times greater than the depth of the multilocus nuclear tree and the time estimate of divergence between *amnicola* and *fasciolata* was 2.5 times older. Similar differences in divergence time estimates based on mtDNA and multilocus nuDNA data are common and have been extrapolated to affect divergences that are more recent than 12 Ma in *Eremophila* larks [32]. The comparison of the divergence time estimates in *Eremophila* mtDNA and multilocus species trees with paleontological record suggested that mtDNA tree estimates were compatible with the currently available paleontological record, whereas the multilocus species tree appeared to underestimate the divergence dates [32]. Unfortunately, we are not aware of any *Locustella* paleontological data except for a single *L*. *naevia* record from the Late Pleistocene of Moravia [33], which is insufficient for determining which tree provides more realistic time estimates.

Due to incomplete resolution within the *certhiola* group in the mtDNA ND2 gene tree, divergence estimates for species from this group could not be compared between the trees. However, on the mtDNA tree the divergence of *amnicola* and *fasciolata* was 1.6 times younger than the initial divergence within *certhiola* species group whereas in the multilocus species tree the split between *amnicola* and *fasciolata* appears to be almost 3 times older than the first divergence within *certhiola* species group.

Substantial introgression of nuclear alleles among all species, the general lack of mtDNA introgression (except the *ochotensis—pleskei* pair and one of 38 *certhiola* carrying *ochotensis* mtDNA), and smaller effective population size of mtDNA relative to nuDNA could be responsible for the lower age estimates of the divergence events in multilocus species tree relative to mtDNA gene tree [10, 34]. However, it cannot explain the relative age differences of divergence events between the *fasciolata* and *certhiola* species groups. Purifying selection and selective sweep of mtDNA in *certhiola* group and apparently neutral evolution of mtDNA in the *fasciolata* group are likely responsible for these relative differences.

### Gene Flow Among Species

The coalescent analysis of gene flow among members of the two species groups based on mtDNA ND2 gene sequences was uninformative since all population size (*θ*) and migration (*M*) parameter values in both species groups had very large confidence intervals that included 0. The only exceptions were the *M* from *ochotensis* to *pleskei* (*M* = 336.8; 95% HPD interval 67.5–967.5) and *θ* for certhiola (*θ* = 0.00293; 95% HPD interval 0.00070–0.00520).

Our multilocus data demonstrated an expected positive association between the range size and *θ* values in both species groups. The population parameter value for widespread continental *fasciolata* was twice that of insular *amnicola*, which has a restricted range. In the *certhiola* species group, continental *certhiola* had a *θ* value almost 3 times that of *ochotensis* inhabiting large islands and Kamchatka, and over 15 times that of *pleskei* which breeds on very small islands.

There was a strong asymmetry in the genetic introgression in all species pairs of both species groups. The migration parameter (*M*) value for the gene flow from *amnicola* to *fasciolata* was almost 7 times greater than the gene flow in the opposite direction. Since the invasion happens in the opposite direction to the apparent gene flow [5, 6], these results suggest that *fasciolata* could have invaded the range of *amnicola*. Perhaps *amnicola* previously inhabited coastal areas of mainland Asia where it was replaced by *fasciolata*.

In the *certhiola* species group, the gene flow from *pleskei* to *ochotensis* and from *ochotensis* to *certhiola* was 3 times greater than gene flow in any other direction. These data suggest that *certhiola* could have invaded the range of *ochotensis*. The ranges of *certhiola* and *ochotensis* overlap along the coast of the Sea of Okhotsk and many individuals with intermediate phenotypes have been reported from that area and neighboring Sakhalin Is. [17]. Interestingly, despite the significant distance (700–800 km) separating ranges of *pleskei* and *ochotensis*, the level of genetic introgression from *pleskei* into *ochotensis* was slightly higher than the level of introgression from *ochotensis* to *certhiola*, whose ranges partially overlap. This suggests that effects of past introgression can still be detected.

## Conclusions

The presence of a strong mito-nuclear conflict in the phylogenetic signal including both the tree topology and divergence time estimates is not a novel finding. Existence of differences in evolutionary histories among loci and dangers of relying on a single locus for inferences about relationships among taxa have been well documented and are long appreciated. Our study, however, joins other recent studies (e.g. [35, 36]) that go beyond documenting such conflicts and attempt to understand the mechanisms creating them and elucidate their effects on phylogenetic reconstruction and species delimitation.

The combination of phylogenetic analyses of mtDNA and multilocus nuDNA sequence data with the coalescent analyses of gene flow among closely related species allowed us to test the effect of genetic introgression and selection on tree topology and divergence date estimates. Consistent with simulations under neutrality and other empirical studies [6], our data showed that nuDNA introgression was substantial and asymmetric among closely related species. As indicated by the direction of these introgressions, species with large ranges appear to have invaded species with smaller ranges. Also consistent with the simulations [6], mtDNA showed no introgression among closely related species except a single species pair where the invader's (*ochotensis*) mtDNA has been swept by the mtDNA of the species it has invaded (*pleskei*). This introgressive sweep of mtDNA had the opposite direction of the nuDNA introgression and resulted in the paraphyly of *pleskei* mtDNA haplotypes with respect to *ochotensis*. Unlike the mtDNA gene tree, the multilocus species tree based on nuDNA data resolved all inter- and intraspecific relationships despite substantial genetic introgression of nuclear loci. However, the node ages on the multilocus species tree may be underestimated due to the effects of substantial genetic introgression [10] as suggested by the differences in node age estimates based of non-introgressing mtDNA and introgressing nuDNA. Finally, introgressive sweep (i.e., positive selection) and strong purifying selection appear to increase internal branch lengths in the mtDNA gene tree. In the mtDNA gene tree, the *certhiola* species group affected by both the introgressive sweep and strong purifying selection was 1.6 times older than the *fasciolata* group where mtDNA evolution is consistent with neutrality. In the multilocus species tree, the *fasciolata* group was almost 3 times older than the *certhiola* group despite the similar levels and patterns of nuDNA introgression in both species groups.

## Supporting Information

S1 Fig TSC [37] networks for individual loci. Only substitutions were treated as differences, gaps were ignored.
(PDF)

S1 Table Individual specimen data (ID, institution, species, sex, date, latitude, longitude, region) and GenBank accession numbers for sequences. (XLSX)

S2 Table Information about loci used in our study (locus ID, genome location, alignment length, forward primer sequence, reverse primer sequence, annealing temperature, references, substitution rate and its 95% HDP intervals, selected substitution model). (XLS)
