# Supplementary figures and images for: Effects of Asymmetric Nuclear Introgression, Introgressive Mitochondrial Sweep, and Purifying Selection on Phylogenetic Reconstruction and Divergence Estimates in the Pacific Clade of Locustella Warblers
Source: PLoS One. 2015 Apr 7;10(4):e0122590. doi: 10.1371/journal.pone.0122590 (PMC4388726; doi:10.1371/journal.pone.0122590)

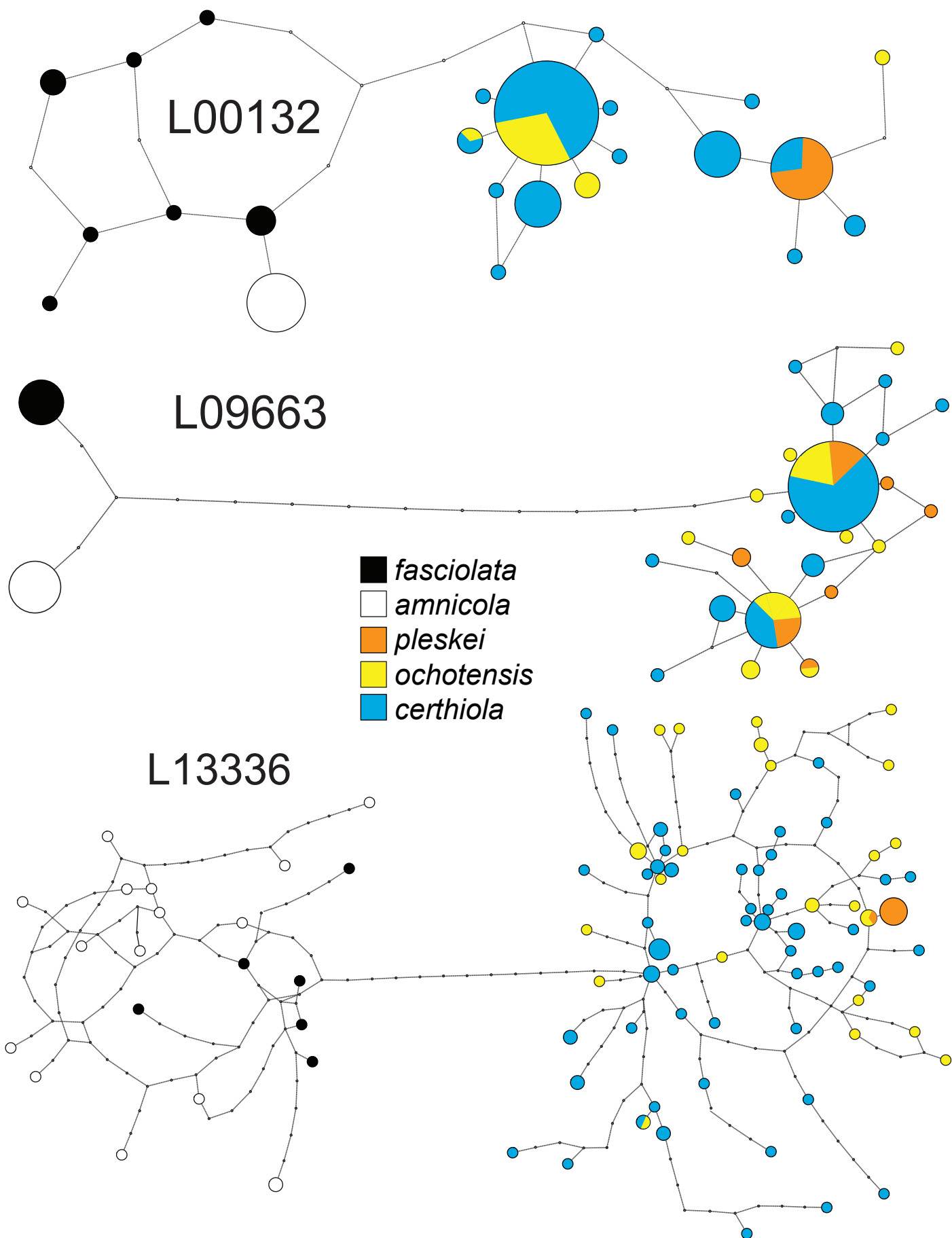

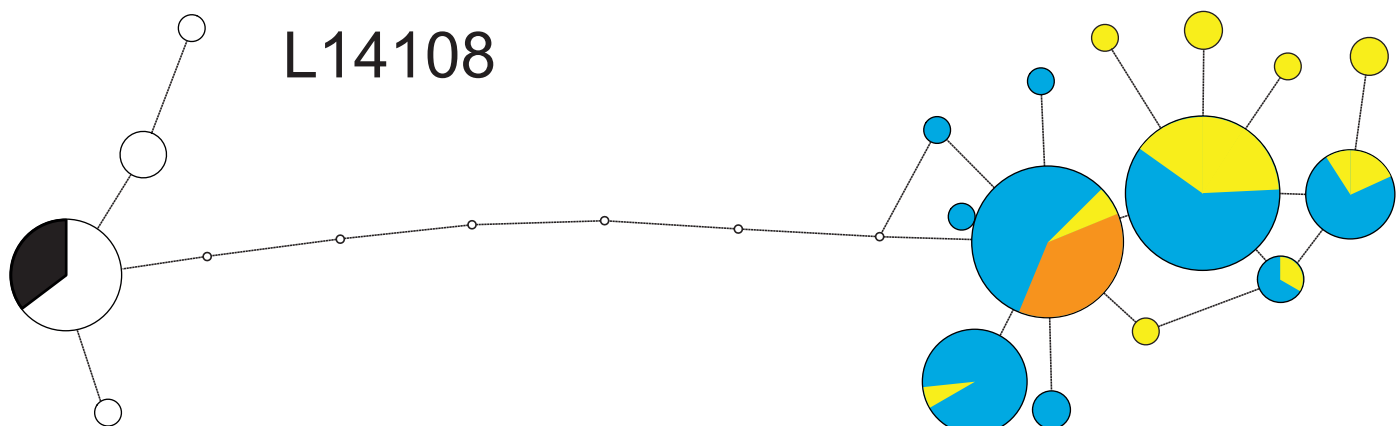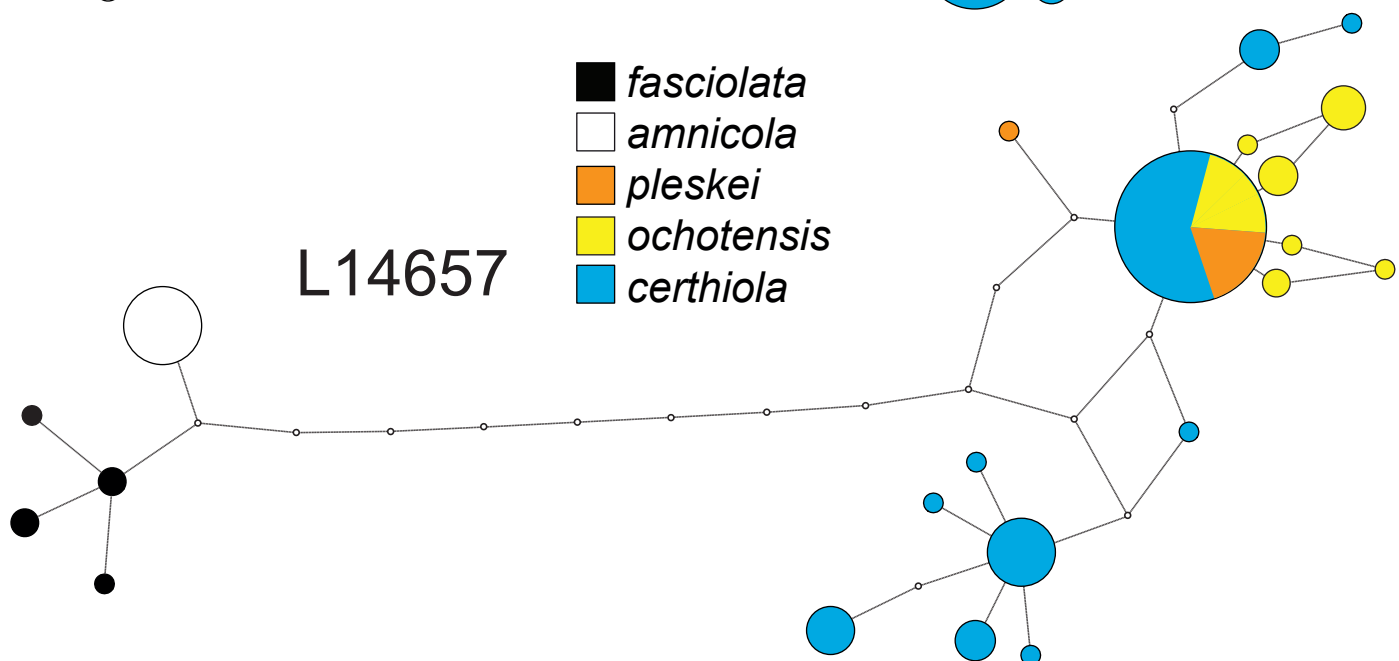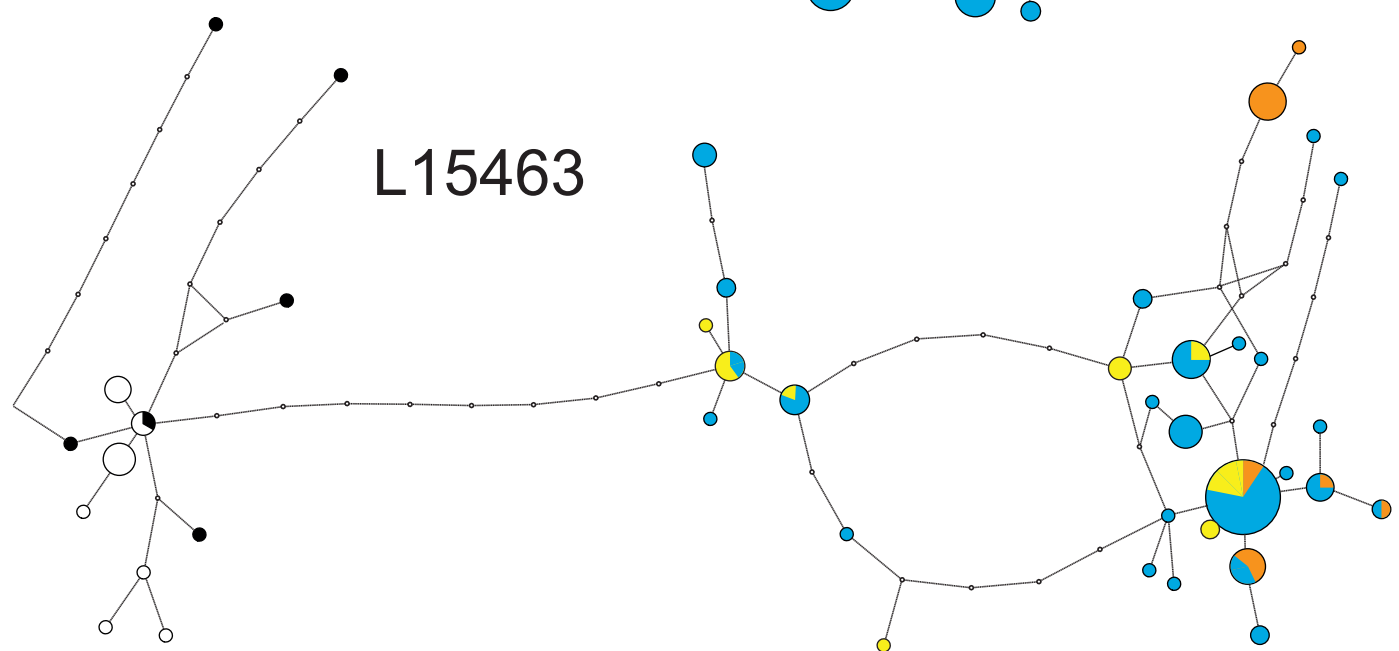

L16532

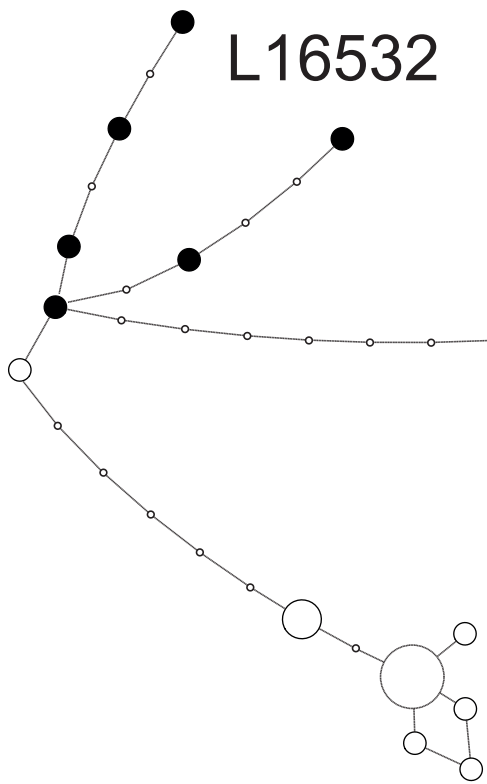

■ *fasciolata*  
□ *amnicola*  
■ *pleskei*  
■ *ochotensis*  
■ *certhiola*

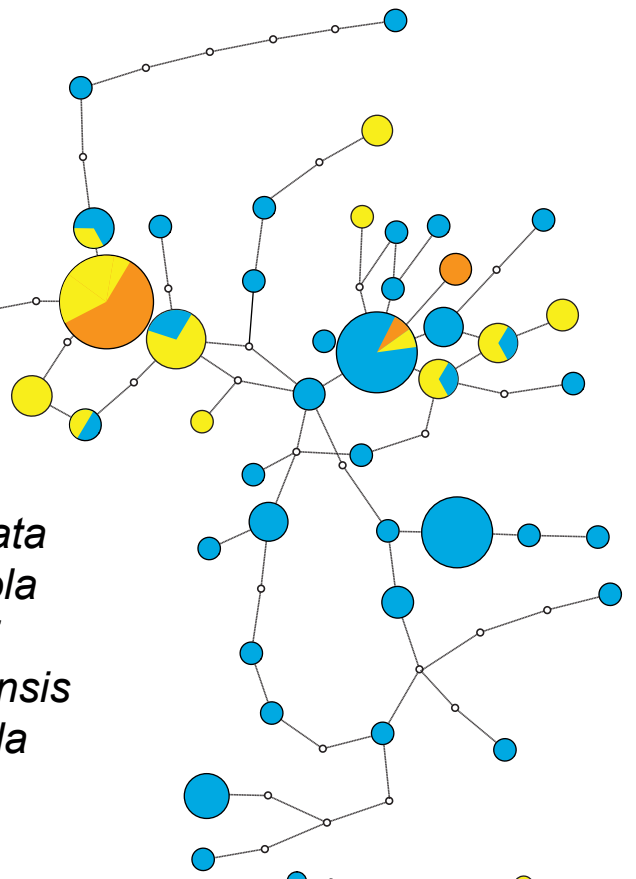

ACL

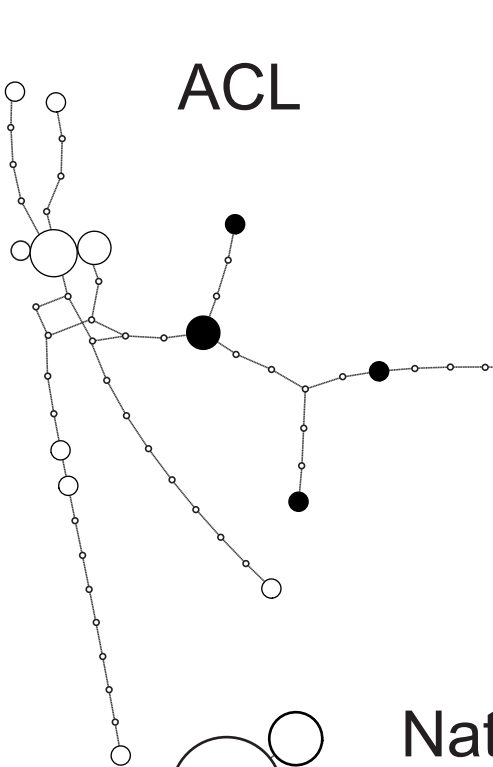

Nat15

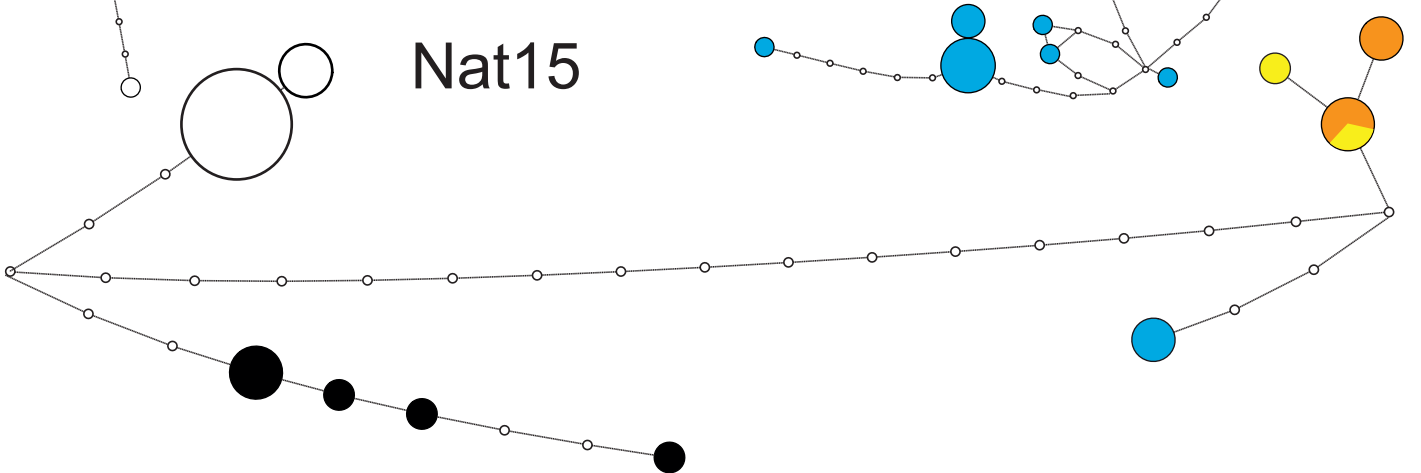

ACO1

RHO

TGBF

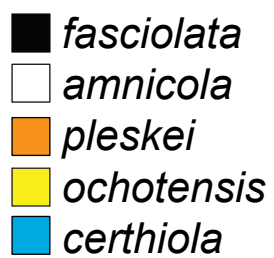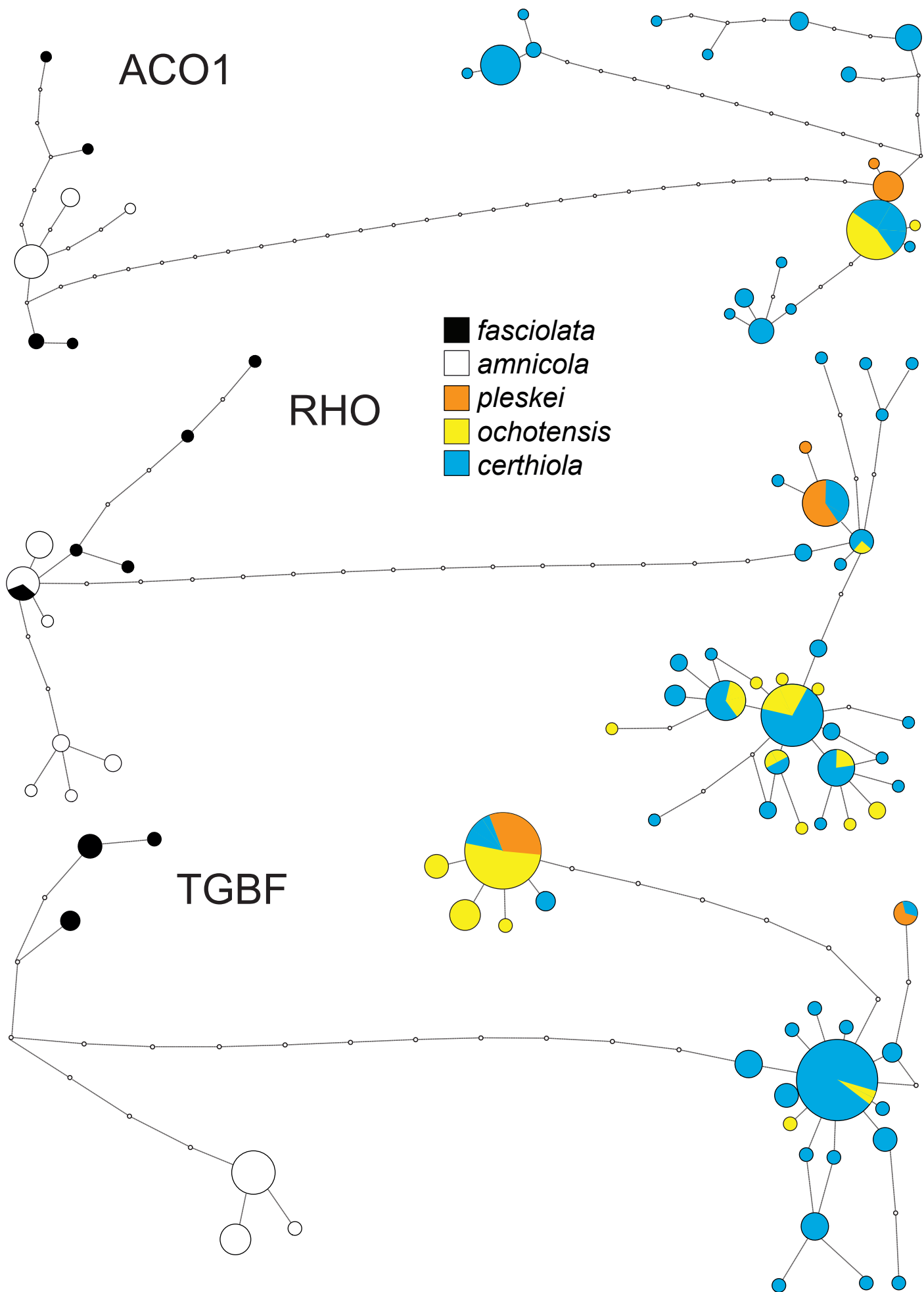

Supplement: S1 Fig — Only substitutions were treated as differences, gaps were ignored. (PDF) [file pone.0122590.s001.pdf]
